# The Ripple Effect: Applying Positive Psychology Principles in Debriefing to Transform Team Culture

**DOI:** 10.7759/cureus.98971

**Published:** 2025-12-11

**Authors:** David O Kessler, Jordyn Feingold, Rachel Elkin, Mirette Dube, Jennifer Reid

**Affiliations:** 1 Emergency Medicine, Columbia University Vagelos College of Physicians and Surgeons, New York City, USA; 2 Psychiatry, Columbia University Vagelos College of Physicians and Surgeons, New York City, USA; 3 Respiratory Medicine, Boston Children's Hospital, Boston, USA; 4 Pediatric Emergency Medicine, Seattle Children's Hospital, Seattle, USA

**Keywords:** behavioral intervention, culture change, healthcare simulation, physician well-being, positive psychology

## Abstract

Healthcare is a complex system filled with high-stress and high-stakes situations that can put workers at risk for burnout and prime negative patterns of thinking. When an individual’s job entails constantly searching for problems or areas of improvement, as in medicine, they can become “stuck” in this paradigm, which may then spill over into social interactions and further compound negative workplace culture. Simulation programs can be effective levers for positive culture change, promoting a growth mindset, learning orientation, and culture of safety. The field of positive psychology can further enhance this by providing specific and explicit tools that help shift individual and team mindsets toward a more positive orientation attuned to what is working well and thereby promote cultures of well-being and resilience. The simulation’s position as an organizational node, with influence that extends beyond the learning environment, makes it uniquely situated to ripple these positive effects through teams, professions, and departments. In this technical report, we explore how core tenets of positive psychology can be leveraged within healthcare settings, where they intersect with concepts already present in debriefing practices, and how to intentionally apply strategies that promote positive workplace culture and individual resiliency. Our goal is to provide a practical guide for integrating positive psychology into simulation and debriefing programs, with a focus on conceptual framing, pragmatic strategies, and implementation tools that support clinician well-being and foster culture change in healthcare.

## Introduction

Modern healthcare systems are often designed with an emphasis on diagnosing illness, evaluating error, reacting to system failures, and debriefing what has gone wrong. While this vigilance is essential for ensuring patient safety, it leaves less room for learning from what goes right. Tools like safety-II, positive event debriefing, and strength-based reflections can broaden our lens toward success, resilience, and system adaptability. Without this balance, the medical model’s deficit orientation, both in theory and in practice, risks eroding clinician morale and fueling burnout [1].

Contemporary researchers have proposed expanding the Institute for Healthcare Improvement's Triple Aim (better health, better care, and lower cost) into a Quadruple Aim by adding a fourth goal: improving the work-life and well-being for the healthcare workforce [2,3]. The Centers for Disease Control and Prevention (CDC) has similarly encouraged integrating professional well-being into all quality improvement initiatives, recognizing healthcare workers as central to the functioning of healthcare systems [4]. Despite increased awareness, particularly in the wake of COVID-19, clinician burnout, moral injury, and psychological distress remain endemic across the health care workforce [5,6]. When a negative mindset dominates healthcare culture, prioritizing error detection over celebrating resilience, clinicians are at risk of accelerated disengagement and loss of meaning in work.

Simulation is uniquely positioned to be a driver of change within organizations [7,8]. Simulation programs are often situated at the junction between the learning environment, quality and safety, interprofessional teamwork, and clinical implementation paradigms, and as such are uniquely positioned to shape behaviors and mindsets. Simulation-based education, particularly through structured debriefing, has long served as a powerful tool for fostering psychological safety, enhancing reflective capacity, and driving positive change in a low-stakes environment. Many of the principles of positive psychology are already embedded in the debriefing pedagogy and lexicon, whether through social learning theory, plus-delta, safety-II/resilience engineering, or growth mindset. These frameworks highlight connection, resilience, and learning from success, values that align closely with key principles of positive psychology.

This article was previously presented in part at a meeting workshop at the 2025 (Munich, Germany) and 2020 annual International Pediatric Simulation Symposia and Workshops meeting for the International Pediatric Simulation Society.

## Technical report

**Figure 1:**
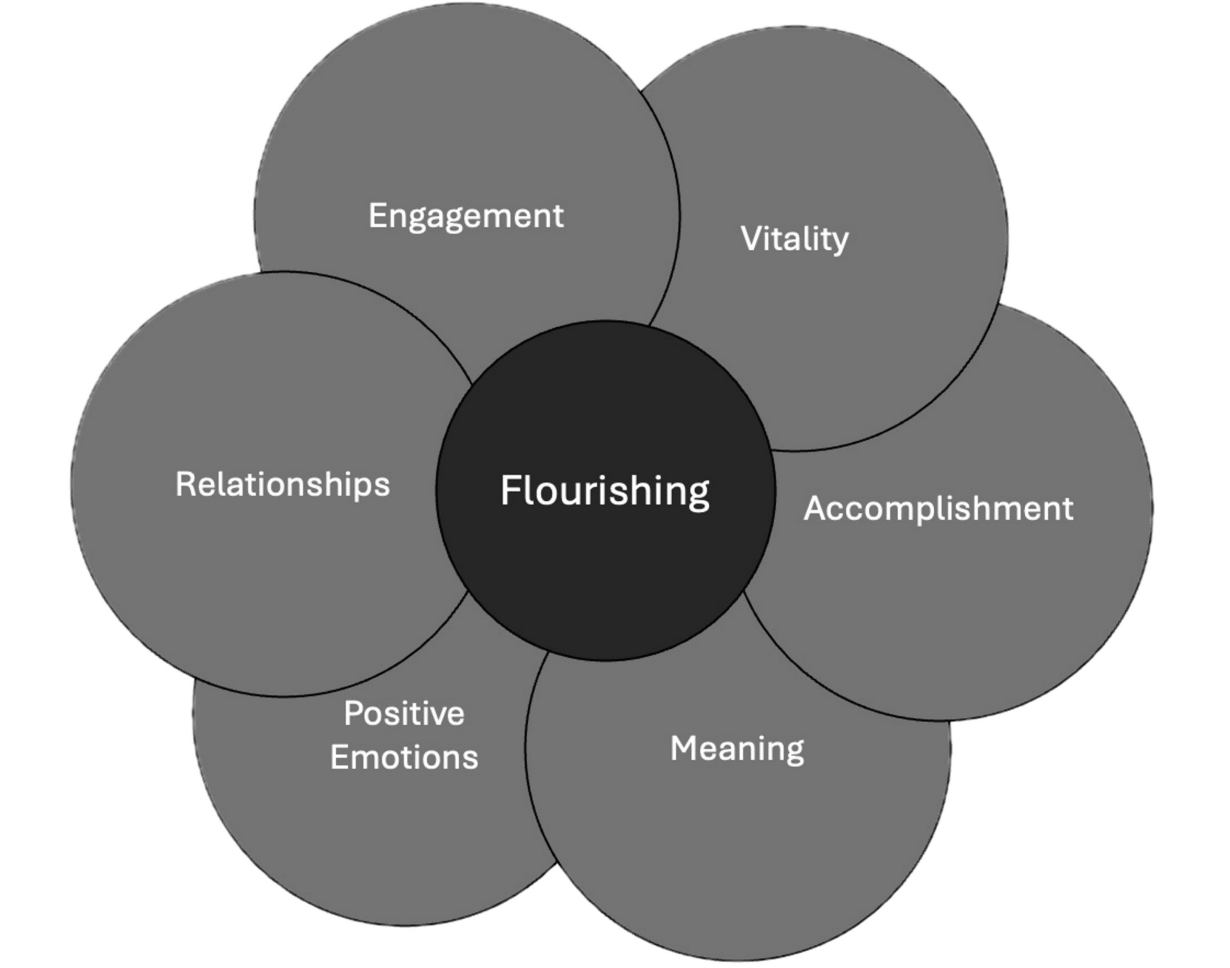
Framework of factors contributing to positive well-being and flourishing (REVAMP) REVAMP stands for relationships, engagement, vitality, accomplishments, meaning, and positive emotions.

Positive psychology, popularized by Martin Seligman [9] at the turn of the 21st century, offers a powerful counterbalance to health care’s prevailing negativity bias. As a subfield of psychology, it focuses on the empirical study of well-being and human flourishing, examining constructs that contribute to individual and collective thriving. Frameworks such as Seligman’s PERMA model (positive emotions, engagement, relationships, meaning, and accomplishment) and the updated REVAMP framework (which adds vitality and is specifically geared for clinical settings) summarize core ingredients of well-being that are especially relevant in healthcare [9,10] (see Figure 1). Many of these concepts already permeate simulation, for example, practicing as a team naturally strengthens relationships at work, yet there are opportunities to be more intentional in how we cultivate them.

Beyond conceptual models, positive psychology researchers have designed and tested intentional activities aimed at cultivating well-being known as positive psychology interventions (PPIs) [11-16] (see Table 1). These interventions target one or more domains of well-being (e.g., meaning, positive emotions, relationships, etc.) and have been shown to enhance resilience, engagement, thriving, and mitigate burnout. Despite a growing body of evidence supporting their effectiveness, positive psychology principles and PPIs have not yet been systematically integrated into the healthcare culture.

**Table 1:** Examples of positive psychology interventions

Positive psychology intervention	Description	Why it works
Gratitude exercises	Discuss three things that went well and why they happened	Encourages noticing positive events, builds positive emotions, counters negativity bias
Best possible self /future self visualization	Imagine what it would look like if everything went as well as possible	Builds optimism and engagement, links to meaning and goals
Use signature strengths in new ways	Identify and use top character strengths	Promotes a growth mindset, builds competence and self-efficacy through positive self-reflection
Acts of kindness	Perform intentional acts of kindness for others	Builds relational well-being, fosters teamwork
Savoring / positive experience recall	Intentionally notice, immerse in, and reflect on positive moments	Amplifies positive emotion, counters negativity bias, and strengthens memory for positive moments
Mindfulness	Intentionally slowing down, increase awareness of the present, notice with all senses, without judgement	Helps with emotional regulation, and bolsters engagement in the present moment
Meaning/values reflection	Reflecting on personal and shared mission, connecting to day-to-day tasks with one's larger sense of purpose in life	Enhances personal and relational engagement in daily life, improves motivation, self-transcendence

The simulation’s impact can be amplified through the intentional and explicit application of positive psychology. This approach is not about suppressing negative emotions, glossing over errors, or avoiding difficult conversations, which risks “toxic positivity” and can undermine psychological safety and trust. Instead, the aim is to acknowledge challenges while framing them in ways that foster growth, resilience, and agency. A balanced approach intentionally draws attention to strengths, progress, and meaning alongside authentic reflection on opportunities for improvement. By incorporating positive psychology frameworks such as the REVAMP model and evidence-based PPIs into debriefing conversations, educators can reframe challenges constructively, reinforce adaptive behaviors, and strengthen social connections and meaning at work (see Table 2). 

**Table 2:** Applying the REVAMP framework to the simulation setting REVAMP stands for relationships, engagement, vitality, accomplishments, meaning, and positive emotions.

REVAMP category	Definition	Possible applications in the simulation	Examples
Relationships	The quality of social connection and trust between team members, perceived support from supervisors	Highlight examples of high-quality teamwork, trust, and collaboration during pre-brief, debrief, or post-simulation communications	Meet with an interprofessional team to plan a simulation case together
			Invite learners to share what team behaviors supported them
			Use plus-delta to capture positive interpersonal dynamics
Engagement	The experience of being fully absorbed, focused, and energized in a task or role	Co-Design immersive, realistic scenarios	Include multiple stakeholders in scenario co-design
			Ask “When did you feel most focused or in the zone?”
			Use video review to highlight moments of high engagement
Vitality	A sense of aliveness that energizes individuals and enables resilience and recovery	Focus on how learners sustained their energy and adapted under stress	Convey gratitude for high energy level
			Normalize discussions of stress responses and discuss adaptive coping
			Format session to enable repeated attempts and opportunity for immediate improvement
Accomplishments	Recognition of progress, mastery, or success in achieving goals	Call attention to individual, team, and system-level improvements resulting from simulation	Ask “What are you most proud of accomplishing today?”
			Document progress across sessions (e.g., faster time to defibrillation)
			Celebrate system improvements (e.g., unsafe conditions identified with simulation)
Meaning	The sense of purpose and significance derived from one’s work and its connection to larger values, such as patient care, teamwork, or service	Connect simulation experiences to broader professional purpose and patient impact	Choose scenarios based on actual patient cases
			Ask if anybody has experienced similar challenges caring for actual patients
			Ask what people will do if they see the same case during their next patient care session
Positive Emotions	Feelings such as joy, gratitude, pride, or relief that broaden thinking, improve coping, and strengthen resilience	Actively elicit pride, joy, or gratitude	Have fun creating novel scenarios or formats (e.g., escape room)
			Open or close debriefing with sharing gratitude
			Highlight humor or human connection where appropriate

Within any healthcare team, there are opportunities to systematically embed positive psychology throughout all stages of the simulation process, from planning and scenario design to debrief and follow-up. Table 3 provides additional examples for the integration of positive psychology across all phases of simulation. 

**Table 3:** Positive psychology intervention opportunities for integration throughout the stages of simulation

Stage	Description	Positive psychology integration opportunity
Needs Assessment and Scenario Planning	Selecting, designing, and preparing the scenario and teaching team	Select learning objectives and cases based on recent, relevant patient scenarios. Involve inter-professional teams to design and teach scenarios - considering relevance for a range of professional roles. Use the planning team as a way to develop relationships and model
Pre-session Communication	All communication (e.g. course notices, emails, announcements, directions, pre-session materials, etc) that participants receive prior to attending the event	“The cases for our session are based on recent patient cases with great outcomes. We will focus on things that have/are going well, so that our entire team can all learn from and integrate these strengths and best practices.”
Facilitator Preparation	Consider the current culture/ outcomes of needs assessment. Identify learning objectives, scripting, logistics (e.g. location, comfort, time, etc) and teaching team structure (e.g. professional roles of debriefers)	Edit current outgoing communication to reflect positive psych principles (see above) Edit current debriefing scripts to reflect positive psychology questions (see below) Look for opportunities in every step to incorporate positive psychology principles
Environment preparation	Everything the participants see, hear, or experience in the simulation space (e.g. welcoming signage, introductory videos, instructions or posted ground rules and assumptions)	Incorporate into non-verbal experience (e.g. Signage “Our events help us to identify our team’s strengths. This depends on the experience and perspective each person brings. Please introduce yourselves to one another and share your role in the team and a goal you have for today’s session”)
Pre-briefing	What is shared/done with the participants prior to the start of the simulation (e.g. clarification of goals, roles, expectations)	Include opportunities for engagement and relationship building. Prior to starting the session, invite participants to participate in a breathing exercise to promote individual calming and mindfulness. Share goals (e.g. “Our goal is to improve how we work together and the care we provide our patients, by identifying our strengths and opportunities for growth).
Simulation scenario	Scenario selection and design including branched phases, based on team actions	Include scenarios with relevance for the team, identify patient care scenarios with successful behaviors/outcomes
Debriefing	Debriefing structure and scripting	Incorporate positive psychology scripting (see Table 4)
Post-session communication	All communication (e.g. learning summaries, issue summaries, mitigation plans) that participants receive after attending the event	“In our last simulation session, we identified two systems issues that are being addressed…We also highlighted individual and group strengths including...”

Thoughtful integration of specific phrases or PPIs during the debriefing phase can further enrich discussion and deepen the impact of positive psychology principles within the experience. While authenticity is paramount, having a few pre-scripted positive prompts or targeted questions, as illustrated in Table 4, can help facilitators apply these principles reliably, especially when they are first getting started. Briefly previewing that you are trying a new technique can also smooth the transition and maintain transparency while building fluency in new techniques. 

**Table 4:** Integrating positive psychology into debriefing

Debriefing phase	Sample positive psychology intervention	Definition	Examples
Setting the scene (create safe context for learning)	Positive introduction	A positive introduction is where somebody introduces a time when they acted virtuously as their “best possible self”	“Take a moment before we begin to think about a moment when you were proudest of yourself or someone else.”
	Positive priming	Positive priming is the intentional activation of positive emotions before an activity	“As we start the debrief, I want you to think of a way you helped during the scenario and also somebody that really helped you.”
Reactions (explore feelings)	Counteract the negativity bias	Intentional focus on positive emotions and reactions can help counterbalance the stickiness of negative emotions	* After initial reactions (all feelings are encouraged, not only positive ones)*: “Those are all valid reactions- now I want us to intentionally identify a moment during the case where you felt that things went smoothly.”
	Mindfulness	Mindfulness is intentional slowing down, increased awareness of the present, noticing with all senses, and without judgment	“As we start to explore our reactions to this case, let’s pause and notice what's going on in each of our bodies (your pulse, your breath, whether you feel calm or nervous. Try to simply notice, without judgment. Would anyone like to share what they are noticing?"
Description (clarify facts)	Connect with meaning	Reflecting on the personal and shared purposes	“Can you summarize the case and share if you have ever had a similar case?” “What did you find most important/relevant about this case?”
	Practice gratitude	Recognizing and appreciating others or systems/ circumstances that support our work and learning	“Can you summarize the case and share if you have ever had a similar case?" "What did you find most important/relevant about this case?”
Analysis (explore performance domains)	Spot character strengths	Identifying and naming character strengths helps promote self-worth, resilience, and well-being. Spotting strengths in team members can build morale and enhance relationships. The values in action (VIA) classification lists 24 character traits that contribute to personality and categorizes strengths into six categories: wisdom, love of learning, courage, humanity, justice, temperance, and transcendence [17]	Facilitators can spot character strengths in group members or invite participants to spot strengths in themselves or each other. Facilitators can share the list of 24-character strengths to provide the language to group members. “I noticed an action that took a lot of courage. [name of participant] I noticed you asked for help with [action]. [name] can you talk to us through your thoughts and actions?” “What did the team notice about the bedside RN during this case, what strengths did he bring to the case?” Groups can also reflect on what strengths they are using collectively in the debrief, e.g. perspective, curiosity, love of learning, bravery, etc.
	Positive reframe	Shifting perspective from a negative interpretation of events to a neutral or positive one (e.g., reframing obstacles as challenges, mistakes as learning opportunities) Considering what values or character strengths are underlying a negative interpretation of events Discuss how negative events may be temporary and isolated, rather than permanent, personal, and pervasive	“You mentioned that moment was tough, what strengths stood out in how the team responded to that challenge?” *In response to a complaint about noise in the room*: “It sounds like you really value a quiet room? How do you like to go about getting quiet” *In response to negative self-talk around a mistake made during the simulation*: “This sounds like a universal challenge. Who else has experienced something like this? How might we view this as something to grow from?”
	Practice Self-Compassion	Treating oneself with kindness, recognizing struggle as universal, and creating healthy emotional distance from painful experiences without disavowing them	*To intervene on a participant’s overly critical self-analyses*: “I'm hearing some hefty self-criticism here. Can I challenge you to think about how you might frame this observation if you were speaking to a dear friend or someone else you really care about after watching them do the same thing?”
Application/Summary (identify take-aways)	Celebrate wins (including growth)	Recognizing and appreciating effective moments, progress, or achievements to reinforce positive emotions and foster a sense of shared meaning	*On top of other learning takeaways*: “Now what’s one thing you’re proud of from this scenario that you’d like to carry forward into your next case or clinical shift?”
	Positive intervention meta-cognition	Explicitly naming positive psychology principles to build awareness, foster a culture of well-being, and encourage a ripple effect for continued practice	“You’ve probably noticed that we intentionally paused today to focus on our unique strengths and share gratitude for each other – these small habits are based on positive psychology principles that help teams and individuals flourish. I hope we’ll carry some of these practices forward into our clinical environment to promote a culture of well-being.”

## Discussion

The following example illustrates a pragmatic approach to integrating positive psychology into practice. 

Needs assessment and planning stage

You are preparing an interprofessional simulation for a clinical unit that has recently experienced rapid growth and turnover. Many team members are early in their careers and motivated by a strong purpose to provide excellent patient care, yet the busy clinical pace has limited opportunities for team reflection and connection during shifts. You want to integrate positive psychology principles but realize that not every intervention can (or should) be applied at once, so to prioritize, you conduct a brief needs assessment talking to a few frontline staff and clinical leaders. Hospital well-being surveys have identified issues of belonging and feelings of isolation and an annual safety attitude questionnaire revealed issues around trust and feeling safe to speak up. Your needs assessment reveals themes of negativity bias (focus on errors), moral distress (from recent bad outcomes amidst resource constraints), burnout (which has eroded vitality), and limited relationships. The fact that frontline staff requested more simulation underscores their strong desire to learn (engagement, meaning, positive emotions), and leadership wants simulation to help disseminate new workflow and space improvements (accomplishments). 

To promote relationships and model collaboration from the outset, you convene a small inter-professional planning group (doctor, nurse, and respiratory therapist). Together, the group decides to prioritize meaning, an existing team strength, and relationships, to build trust and support across the team. They select a recent case with strong clinical relevance and emotional resonance, and design a scenario that offers shared problem-solving opportunities for all team members.

Pre-simulation stage

In pre-session communication, you share pride in the team’s inter-professional approach to planning the simulation experience. You acknowledge the time and effort that both planners and participants are investing and express gratitude for their engagement. In highlighting the purpose of the simulation, you emphasize the incorporation of key points from recent meaningful cases and highlight the recent accomplishments of newly rolled out resources in the room being used.

On the day of the session, you welcome people with theme music from a popular action movie to energize people and make them laugh as they come in, employing fun and playfulness to promote feelings of vitality and connection. At the entrance to the simulation room, there is a brief message of gratitude on the sign-in log and a QR code with a link for the positive quote of the day. Learners are asked to silence phones to support presence and mindfulness. Name badges are used to encourage introductions between participants. 

During the pre-brief, learners introduce themselves and share one thing they are proud about from the prior week. Before beginning, you guide the team in a brief mindful moment where they close their eyes, breathe in together and then slowly breathe out while focusing on the intention to take risks together during the simulation to foster learning. 

Simulation stage

The simulation scenario carefully blends elements from recent clinical cases that are strongly relevant for all members of the inter-professional team. Branch points in the case are designed to be triggered by specific aspects of teamwork and communication. It is intentionally written to provide adequate challenge and reinforce a sense of accomplishment with completion of challenging tasks while eliciting meaningful opportunities for reflection on improvement. The quality of chest compressions (depth, rate, and recoil) is recorded by a high-technology manikin to facilitate discussion about quality and launch of a new mock code chest compression leaderboard (something the team came up with as a motivator) that enhancing engagement by posting high scores in the break room every month. 

Debriefing stage

After completing the scenario, your planning team helps the team transition to a learning stance by having them step away from the simulation space, take three deep breaths (mindfulness), close their eyes, and picture one thing they saw or heard another teammate do well during the case (positive experience recall and priming). 

You start the debrief using the PEARLS (Promoting Excellence and Reflection in Learning) debriefing framework [18], actively incorporating positive psychology principles and interventions throughout the five phases of the debrief (setting the scene, reactions, description, analysis, and summary). Co-debriefers actively invite reactions from each professional group, acknowledging expertise and strengths across roles, and model mutual respect in the discussion. Throughout the debrief, you weave in principles of meaning and relationships, prompting reflections with inquiries such as: “What did you find most important or relevant about this case?" and "Can someone share a few things you're grateful for, big or small, about how the team worked together?" Pre-scripting some positive prompts or targeted questions based on examples in Table 4 helps ensure these principles are reinforced consistently.

Post-simulation stage

After the simulation, you complete the learning cycle by following up with an email to the participants and take home points for the rest of the unit. You once again convey gratitude to the team and leadership for their engagement and commitment to improving care and growing together as a team. The connection to real clinical cases and reinforcement of new work resources and processes underscore the relevance of the session and its direct link to process improvement. While preserving anonymity (and thereby psychological safety), small wins and accomplishments can be called out. With the team’s prior permission a shout-out is made for the winner of the new “SimCompression leaderboard," highlighting accomplishments and the shared purpose of commitment to excellence.

## Conclusions

In a time when healthcare teams are facing systemic challenges to well-being, positive psychology offers a practical framework for simulation-based interventions that strengthen relationships, engagement, vitality, meaning, accomplishment, and positive emotions at work. By intentionally embedding practices such as gratitude, mindfulness, strengths recognition, and positive reframes into debriefing, simulationists can help foster individual and team well-being. Positive psychology approaches are not designed to silence or ignore negative experiences. It is important to avoid “toxic positivity” and integrate interventions along with authentic reflections on error, stress, and system challenges to maintain psychological safety. A blended approach to learning from errors while reinforcing a positive mindset helps promote an orientation towards continued growth and sense of purpose. 

Looking ahead, simulation may serve not only as an optimal environment for rehearsing clinical performance, but also as a space for processing system failures, moral distress, and emotional burden. Deliberately integrating team simulation and clinical debriefing into organizational well-being strategies could provide fertile ground for innovation in applied positive psychology interventions. Ultimately, the goal is to translate individual well-being to system-level culture change and improved clinical outcomes.
